# Machine learning–couched treatment algorithms tailored to individualized profile of patients with primary anterior chamber angle closure predisposed to the glaucomatous optic neuropathy

**DOI:** 10.1007/s13167-023-00337-1

**Published:** 2023-08-17

**Authors:** Natalia I. Kurysheva, Oxana Y. Rodionova, Alexey L. Pomerantsev, Galina A. Sharova, Olga Golubnitschaja

**Affiliations:** 1 The Ophthalmological Center of the Federal Medical and Biological Agency of the Russian Federation, 15 Gamalei Street, Moscow, Russian Federation 123098; 2 Federal Research Center for Chemical Physics RAS, 4, Kosygin Street, Moscow, Russian Federation 119991; 3 Ophthalmology Clinic of Dr. Belikova, 26/2, Budenny Avenue, Moscow, Russian Federation 105118; 4 Predictive, Preventive and Personalised (3P) Medicine, Department of Radiation Oncology, University Hospital Bonn, Rheinische Friedrich-Wilhelms-Universität Bonn, 53127 Bonn, Germany

**Keywords:** Predictive preventive personalized medicine (PPPM / 3PM), Treatment modalities, Laser peripheral iridotomy, Lens extraction, Multi-parameter analysis, Machine learning, Artificial intelligence, Individualized patient profile, Phenotyping, Primary care, Primary open-angle glaucoma, Improved individual outcomes, Health policy

## Abstract

**Background:**

Primary angle closure glaucoma (PACG) is still one of the leading causes of irreversible blindness, with a trend towards an increase in the number of patients to 32.04 million by 2040, an increase of 58.4% compared with 2013. Health risk assessment based on multi-level diagnostics and machine learning–couched treatment algorithms tailored to individualized profile of patients with primary anterior chamber angle closure are considered essential tools to reverse the trend and protect vulnerable subpopulations against health-to-disease progression.

**Aim:**

To develop a methodology for personalized choice of an effective method of primary angle closure (PAC) treatment based on comparing the prognosis of intraocular pressure (IOP) changes due to laser peripheral iridotomy (LPI) or lens extraction (LE).

**Methods:**

The multi-parametric data analysis was used to develop models predicting individual outcomes of the primary angle closure (PAC) treatment with LPI and LE. For doing this, we suggested a positive dynamics in the intraocular pressure (IOP) after treatment, as the objective measure of a successful treatment. Thirty-seven anatomical parameters have been considered by applying artificial intelligence to the prospective study on 30 (LE) + 30 (LPI) patients with PAC.

**Results and data interpretation in the framework of 3P medicine:**

Based on the anatomical and topographic features of the patients with PAC, mathematical models have been developed that provide a personalized choice of LE or LPI in the treatment. Multi-level diagnostics is the key tool in the overall advanced approach. To this end, for the future application of AI in the area, it is strongly recommended to consider the following:

1. Clinically relevant phenotyping applicable to advanced population screening
2. Systemic effects causing suboptimal health conditions considered in order to cost-effectively protect affected individuals against health-to-disease transition
3. Clinically relevant health risk assessment utilizing health/disease-specific molecular patterns detectable in body fluids with high predictive power such as a comprehensive tear fluid analysis.

**Supplementary Information:**

The online version contains supplementary material available at 10.1007/s13167-023-00337-1.

## Background

### Risk factors of glaucomatous optic neuropathy relevant for primary care

Primary angle closure glaucoma (PACG) is still one of the leading causes of irreversible blindness, with a trend towards an increase in the number of patients to 32.04 million by 2040, an increase of 58.4% compared with 2013 [1]. In the case of PACG, the risk of developing bilateral blindness is three times higher than in primary open-angle glaucoma (POAG) [2]. From this point of view, the early detection of angle closure suspects is of high importance. While PACG patients with previous symptomatic angle closure demonstrate mild or moderate VF defects at the time of the first presentation to an ophthalmologist, patients with asymptomatic PACG often have the end-stage of the disease [3].

The progression rate is particularly high in primary angle closure suspects (PACS) [4]. Thomas et al. [5] analyzed the results of a 5-year follow-up of patients with PACS and noted the transition to primary angle closure (PAC) in 22% of cases; the rate of progression was 4.4% per year. Wilensky et al. [6] found a similar rate of progression of PACS: in 19.4% of cases (25 patients out of 129), a transition to PAC was recorded in 2.7 years, including 8 (6.2%) cases that developed an acute attack. Decade observation of Alsbirk [7] demonstrated the progression of PACS to PAC/PACG among Greenlandic Eskimos in 35% of cases, the rate of progression was 3.5% per year. As part of the randomized ZAP study (The Zhongshan Angle Closure Prevention Trial), patients with bilateral PACS were followed up, where laser peripheral iridotomy (LPI) was performed on one eye, and the fellow eye remained intact [8]. After 6 years, progression in eyes that underwent laser intervention was detected in 2% of cases, and in paired eyes, in 4% of cases. Adjusted for age, gender, and baseline intraocular pressure (IOP), the risk of PACS progression was 0.52 (hazard ratio (HR) 0.52; 95% confidence interval (CI) 0.30–0.91; *p* = 0.023). It was concluded that in order to prevent one case of PACS progression, it is necessary to perform LPI in 44 eyes, which does not seem appropriate from a practical and economical point of view.

The issue of optic neuropathy in PACG progression is relevant in terms of the burden on the healthcare system, the economic component, and the quality of life of patients. It is important to search for predictors of PAC to prevent the development of glaucomatous optic neuropathy (GON). The question of in which cases PACS will move to PAC, and then to PACG, remains open. In the course of numerous studies, the authors propose various predictors of the development of early stages of primary angle closure disease (PACD): increased iris volume [9] and lens vault (LV) [10], age over 59 years, decreased angle opening distance of 500 μm from the scleral spur, and increased curvature of the iris [11].

Eyes with a large peripheral anterior synechia (PAS) extent are at higher risk for progression and are also more likely to have associated GON [5, 12].

Progression of PACG depends not only on the parameters of the anterior chamber, but also on race [13]. It is assumed that this fact is associated with different mechanisms in the development of occlusal angles. Iris thickness has been shown to be an important predictor of occlusal angles in Caucasians but not in ethnic Chinese, and lens vault is an important parameter in anterior segment optical coherence tomography (AS-OCT) in screening for angle closure in Caucasians [14]. In fact, angle closure occurs due to various mechanisms including relative pupillary block, iris configuration (plateau syndrome, thick iris, anterior position of the ciliary body), and lens-related mechanisms (thick lens, anterior position), as well as, possibly, effusion of the choroid. At the same time, several mechanisms can contribute to angle closure in one eye, and each mechanism contributes differently to the development of the disease in different eyes [15]. It should be noted that the presence of several mechanisms of blockade of the anterior chamber angle (ACA) dictates a personalized approach to the treatment of PACS.

The relevance of the treatment of PACD in the early stages before the formation of GON is recognized by many researchers [16–20]. However, the question concerning the method of choice for PAC treatment (LPI or lens extraction, LE) remains still open [21]. Obviously, the answer to this question is associated with the personalized medicine approach.

### Multi-parametric analysis is essential to promote personalized medical services to patients diagnosed with PACD

To determine a more effective method of treatment, multiple data analysis is required, reflecting the individual characteristics of the patient. Such an analysis makes it possible to identify the mechanism of closing the anterior chamber angle more accurately. Pupillary block is the leading cause of closed angles in Western countries [22]. It is characterized by an iridotrabecular contact, increased curvature of the iris, and a shallow anterior chamber. LPI is considered an effective treatment for pupillary block [23]. Conversely, the plateau iris configuration and anterior ciliary body configuration characteristic of Asian populations are associated with normal or slightly reduced anterior chamber depth (ACD) [24]. In this case, LPI is not effective and laser peripheral iridoplasty is recommended [25]. The lens enlargement is characterized by its high arch, anterior position relative to the axial length (AL), which leads to a “volcano-like” configuration of the iris. In this case, lens extraction is appropriate [26]. “Posterior-lenticular” causes of PAC are associated with an increase in the thickness of the choroid [27]. In a study by Kurysheva et al. [28], more than 37 clinical and anatomical parameters of each patient with PAC were taken into account for a personalized treatment method.

### Application of artificial intelligence in ophthalmology: machine learning approach in PACD services

Most studies on glaucoma are based on the use of mechanistic models [29–33]. Nowadays, there are great hopes for the use of a wide range of methods of machine learning (ML)–artificial intelligence (AI) as a tool for diagnosis, choice of treatments, and prediction of outcomes for PACD. A detailed review can be found here [34]. It is well known that ML methods primarily rely on a formal approach and application of big datasets. At the same time, a combination of a formal AI approach together with a careful selection of representative groups of patients [35] and principles of physiology provide the so-called ML physiology-informed methods [36].

Various ML methods have been recently applied in the diagnosis of glaucoma [37, 38] and for the detection of primary angle closure suspects based on static and dynamic anterior segment parameters [39].

In order to reduce the burden on primary care, research is being conducted aimed at automatically assessing the parameters of the anterior chamber of the eye using imaging methods such as AS-OCT to detect angle closure [40–42].

An important guideline for quantifying ACA is the scleral spur [43]. Machine learning methods are used to localize the scleral spur for measurement of the ACA in the clinical evaluation of angle closure [44, 45]. Liu et al. [45] demonstrated that deep learning (DL) model is able to locate the scleral spur in AS-OCT images with high repeatability. These new technologies may help advance risk assessment of angle closure eyes.

Hao et al. [46] used neural networks for differential diagnosis of appositional and synechial angles under light–dark on AS-OCT conditions, similar to dynamic gonioscopy. Despite the fact that the model obtained by the authors currently erroneously classifies the synechial angle as an appositional angle, the study helps the doctor in making a diagnosis and monitoring. Certainly, further research in this direction is required.

Also, Wang et al. [47] showed that a deep learning system could automatically detect angle closure and quantitatively measure angle parameters from ultrasound biomicroscopy (UBM) images and enhancing the intelligent diagnosis and management of PACG.

Anterior chamber depth values are known to be used to screen for angle closure. DL predicts ACD from low-cost anterior segment photographs on par with an ocular biometer and AS-OCT [48].

Neural networks can be used to detect angle closure in goniophotographs with performance comparable to that of an experienced glaucoma specialist [49].

Despite the successful use of DL in PACD, there are a number of problems. Generalizable DL models require large amounts of diverse and well-labeled data generated by experts, which is time-consuming and suffers from inherent interrater variability [50]. Zheng et al. [51] used generative adversarial networks to create new medical images that serve as training datasets for the development of DL algorithms for the detection of angle closure. Applying synthesized new images will improve DL learning models to reliably classify ACA.

## Working hypothesis in framework of 3P medicine

The present study was initiated due to the lack of literature on the use of mathematical modelling methods based on machine learning in the field of assessing the efficacy of PAC treatment. We hypothesized that the multivariate data analysis utilizing machine learning may lead to the development of clinically applicable models relevant to improved individual outcomes of the primary angle closure treatment by predicting and preventing glaucomatous damage. For doing this, we suggested a positive dynamics in the intraocular pressure (IOP) after treatment, as the objective measure of a successful treatment. Thirty-seven anatomical parameters have been considered by applying artificial intelligence to the prospective study on 30 (LE) + 30 (LPI) patients with PAC.

The proposed approach follows principles of the paradigm change from reactive medical services (applied to clinically established glaucomatous damage) to predictive, preventive, and personalized medicine (3PM/PPPM) applied to vulnerable groups in the population. Great impacts are expected by improving individual outcomes of preventable glaucomatous damage (concretely PACG) accompanied by positive cost-efficacy of advanced medical services to the population (e.g., in the form of innovative screening programs) utilizing predictive disease modelling and treatment algorithms tailored to the personalized patient profile [52]. Essential multi-parametric analysis is implementable by utilizing artificial intelligence (machine learning) in the area [53].

## Study design

### Clinical approach

The study was performed in accordance with the ethical principles specified in the Declaration of Helsinki, the Good Clinical Practice (GCP), and regulatory requirements. The study included 76 Caucasian patients aged 41 to 80 years, examined from January 2019 to December 2021. All patients gave their informed consent to participate in the study.

#### Inclusion criteria

PAC patients with IOP up to 30 mmHg. All study participants had a spherical equivalent of − 6.0 D to + 6.0 D and astigmatism up to 2.0 D. The diagnosis of PAC was established if the posterior pigmented part of the trabecular meshwork was not visible in gonioscopy for more than 180° at the primary position without GON, but in combination with increased IOP and/or with the presence of PAS [54]. The study included patients with a transparent lens, or with initial opacities according to the LOCS III classification (Lens Opacities Classification System) in the nucleus up to NC2 (Nuclear Color/Opalescence) and/or in the cortex up to C2 (Cortical) and/or along the posterior capsule up to P2 (Posterior Subcapsular) based on biomicroscopy data [55].

#### Exclusion criteria

Included the lack of stable fixation, eye surgery in previous medical history, including laser surgeries, the presence of chronic systemic autoimmune diseases, diabetes mellitus, Parkinson’s disease, Alzheimer’s disease, and dementia. We did not include patients with a pupil diameter < 3.0 mm under mesopic conditions according to optical biometrics (AL-Scan, NIDEK, Japan), as well as patients using miotic agents. Low preoperative corneal endothelial cell count (ECC) (< 1000 cells/mm^2^) was also considered an exclusion criterion.

Patients with primary angle closure who voluntarily consented to the medical intervention were randomly assigned to two equally sized groups, which were then tested for similarity. The first group (30 eyes) included PAC patients who underwent LE. The second group (30 eyes) included PAC patients who underwent LPI.

All patients underwent the following procedures before the treatment and 4 weeks after LE and LPI: autorefractometry (autorefractometer RT-5100, NIDEK, Japan), visometry (chart panel CP-770, NIDEK, Japan), gonioscopy (goniolens VG4LNF, VOLK, USA), optical biometry (AL-Scan, NIDEK, Japan), static automatic perimetry (SAP) (Humphrey Field Analyzer HFA-II 750i, Carl Zeiss, Germany, SITA Standard 24–2), biomicroscopy (slit lamp SL 1800, NIDEK, Japan), corneal confocal microscopy (ConfoScan 4, Nidek, Japan), ophthalmoscopy (non-contact lens 90 D, Volk Optical, USA), and swept-source optical coherence tomography (SS-OCT) of the posterior and anterior sections (Revo NX130, Optopol, Poland). IOP was measured by Goldmann applanation tonometry. IOP was measured from 10 a.m. to 12 a.m. Gonioscopy was performed in a dark room with a patient at the primary position. The ACA opening was assessed in all quadrants according to Schaffer’s scale. Compression gonioscopy was performed to evaluate the presence of peripheral anterior goniosynechia. The presence/absence of GON was determined on the basis of SAP, SS-OCT, and ophthalmoscopy data. PAC and PACS eyes should have no evidence of GON (vertical cup-to-disc ratio (CDR) ≥ 0.7 and/or asymmetric CDR > 0.2 and/or focal notching) and compatible visual field loss on Humphrey perimetry.

The ACA parameters were measured using SS-OCT with the anterior segment module (AS-OCT) [56]. Only the images with a quality index (QI) above 8 were analyzed.

The *macular choroidal thickness* (CT) was measured in horizontal and vertical directions according to the previously described method [57].

### Laser peripheral iridotomy and lensectomy with intraocular lens implantation

LPI was performed using Optimis II YAG laser (Quantel Medical, France) according to the standard method with an Abraham lens (Ocular Instruments, Bellevue, WA, USA) [8].

Phacoemulsification with the implantation of a monofocal or multifocal intraocular lens (IOL) was performed in accordance with the target refraction to achieve an optimal anatomical and reconstructive effect.

The treatment hypotensive effect was understood as an IOP decrease (ΔIOP) after the intervention relative to the baseline.

### Machine learning approach

The machine learning methods are based on the analysis of multivariate data. We used a one-class classifier and principal component regression [58–60].

To confirm the similarity between groups before LE (LE_pre) and before LPI (LPI_pre), a one-class classifier approach [61] was used. The chosen classifier is Data-Driven Soft Independent Modelling of Class Analogies (DD-SIMCA) which description can be found in the literature [62].

To develop the regression models to predict the hypotensive effect of PAC treatment of the anterior chamber, the method of principal component regression (PCR) was applied [58]. The predictor matrix **X** consists of data of clinical and anatomo-topographic features obtained before the treatments (Table 1).

**Table 1:** Pre- and post-LE, pre- and post-LPI parameters in PAC; note: the table shows the mean values and standard deviation; pre-LE—patients before lens extraction (LE); post-LE—patients after LE; pre-LPI—patients before laser peripheral iridotomy (LPI); post-LPI—patients after LPI; PAS—peripheral anterior synechiae; *p-value between pre-LE and post-LE; **p-value between pre-LPI and post-LPI; ***p-value between LE-pre and pre-LPI; ****p-value between post-LE and post-LPI; ^the LOCS III standards (Lens Opacities Classification System); ^^number of eyes; *AOD* angle opening distance; *TISA* trabecular-iris space area; ^§^*p* value according to Wilcoxon for dependent sample; ^δ^*p* value according to Mann–Whitney for independent samples; the absolute value of the eyes is given in parentheses; *p*-value < 0.05 are indicated in bold; ^a^evaluation of the anterior chamber angle was based on the Shaffer gonioscopic grading classification: an angle between the iris and the trabecular meshwork surface of 35° to 45° was classified as grade 4, between 20° and 35° was classified as grade 3, between 10° to 20° was classified as grade 2, and less than 10° was classified as grade 1. Grade 0 was assigned if angle structures were not observed

Characteristic	Pre-LE (*n* = 30)	*p*-value**	Post-LE (*n* = 30)	Pre-LPI (*n* = 30)	*p*-value *	Post-LPI (*n* = 30)	*p*-value***	*p*-value****
Age, years	64.1 ± 11.4	62.6 ± 9.9	0.487^δ^	–				
Gender (male/female)	11/19	10/20	–	–				
Lens opacity (grade N01–NC2, C1–C2, P1–P2)^	47% (14^^)	37% (11^^)	0.436	–				
IOP, mm Hg	25.5 ± 2.3	**0.000**^§^	17.2 ± 1.19	24.6 ± 2.1	**0.000**^§^	19.7 ± 0.8	0.765^**δ**^	**0.000**^**δ**^
Glaucoma topical medication mean (SD)	0.63 ± 0.49	**0.000**^§^	0.07 ± 0.25	0.60 ± 0.50	0.317^§^	0.43 ± 0.50	0.792^**δ**^	**0.001**^**δ**^
ΔIOP, mm Hg	–	–	7.33 ± 3.40	–	–	4.87 ± 2.42	–	**0.016**^**δ**^
Spherical equivalent, *D*	1.53 ± 1.38	**0.000**^**§**^	− 0.07 ± 0.16	1.65 ± 1.12	0.317^§^	1.63 ± 1.10	0.888^**δ**^	**0.000**^**δ**^
Uncorrected visual acuity, UVA	0.17 ± 0.17	**0.000**^**§**^	0.95 ± 0.08	0.31 ± 0.23	0.317^§^	0.31 ± 0.23	**0.005**^**δ**^	**0.000**^**δ**^
Best-corrected visual acuity, BCVA	0.89 ± 0.18	**0.003**^**§**^	0.98 ± 0.05	0.92 ± 0.13	1.0^§^	0.92 ± 0.13	0.564^**δ**^	**0.030**^**δ**^
Shaffer angle opening degree^a^ on 90^0^	0.61 ± 0.52	**0.000**^§^	2.93 ± 0.25	0.63 ± 0.49	**0.000**^§^	1.93 ± 0.25	0.894^**δ**^	**0.000**^**δ**^
Shaffer angle opening degree^a^ on 270°	0.72 ± 0.49	**0.000**^§^	3.0 ± 0.37	0.73 ± 0.45	**0.000**^§^	2.07 ± 0.25	0.874^**δ**^	**0.000**^**δ**^
AOD500_90°, mm	0.061 ± 0.019	**0.000**^**§**^	0.338 ± 0.063	0.063 ± 0.018	**0.000**^§^	0.179 ± 0.019	0.877^**δ**^	**0.000**^**δ**^
AOD750_90°, mm	0.115 ± 0.048	**0.000**^**§**^	0.500 ± 0.075	0.116 ± 0.046	**0.000**^§^	0.236 ± 0.036	0.871^**δ**^	**0.000**^**δ**^
TISA500 _90°, mm^2^	0.024 ± 0.006	**0.000**^**§**^	0.120 ± 0.021	0.025 ± 0.005	**0.000**^§^	0.058 ± 0.006	0.877^**δ**^	**0.000**^**δ**^
TISA750_90°, mm^2^	0.047 ± 0.015	**0.000**^**§**^	0.224 ± 0.037	0.048 ± 0.013	**0.000**^§^	0.112 ± 0.013	0.832^**δ**^	**0.000**^**δ**^
AOD500 _270°, mm	0.078 ± 0.031	**0.000**^**§**^	0.364 ± 0.069	0.079 ± 0.029	**0.000**^§^	0.201 ± 0.117	0.784^**δ**^	**0.000**^**δ**^
AOD750_270°, mm	0.137 ± 0.061	**0.000**^**§**^	0.540 ± 0.094	0.131 ± 0.061	**0.000**^§^	0.284 ± 0.021	0.600^**δ**^	**0.000**^**δ**^
TISA500_270°, mm^2^	0.027 ± 0.009	**0.000**^**§**^	0.131 ± 0.025	0.028 ± 0.008	**0.000**^§^	0.064 ± 0.005	0.487^**δ**^	**0.000**^**δ**^
TISA750_270°, mm^2^	0.054 ± 0.021	**0.000**^**§**^	0.245 ± 0.044	0.055 ± 0.019	**0.000**^§^	0.124 ± 0.009	0.871^**δ**^	**0.000**^**δ**^
Presence of PAS, *N* (%)	13% (4^^)		10% (3^^)	13% (4^^)	–	13% (4^^)	–	–
Anterior chamber depth, ACD	2.33 ± 0.26	**0.000**^§^	3.63 ± 0.199	2.34 ± 0.28	**0.000**^§^	2.36 ± 0.280	0.877^**δ**^	**0.000**^**δ**^
Lens vault, LV, mm	0.866 ± 0.155	–	–	0.864 ± 0.120	**0.000**^§^	0.843 ± 0.110	0.918^**δ**^	–
Iris Curvature, ICurv nasal, mm	0.316 ± 0.087	**0.000**^**§**^	0.162 ± 0.407	0.319 ± 0.076	**0.000**^§^	0.225 ± 0.042	0.723^**δ**^	**0.000**^**δ**^
Iris Curvature, ICurv temporal, mm	0.317 ± 0.087	**0.000**^§^	0.163 ± 0.035	0.320 ± 0.078	**0.000**^§^	0.224 ± 0.044	0.734^**δ**^	**0.000**^**δ**^
Iris thickness, IT750 in the nasal sector, mm	0.406 ± 0.047	**0.000**^§^	0.400 ± 0.050	0.404 ± 0.046	0.221^§^	0.403 ± 0.047	0.871^**δ**^	0.701^**δ**^
IT750 in the temporal sector, mm	0.407 ± 0.049	**0.000**^§^	0.399 ± 0.052	0.404 ± 0.046	0.157^§^	0.404 ± 0.047	0.842^**δ**^	0.626^**δ**^

The response vector **y** includes the values calculated as the difference between IOP obtained before and after the treatment: ΔIOP = IOP_pre_ − IOP_post_. The models’ validation was done using the Procrustes cross-validation (PCV) method [63]. The performance was evaluated using the root mean squared error

$$RMSE=\sqrt{\frac{1}{I}\sum_{i=1}^{I}{\left({y}_{i}-{\widehat{y}}_{i}\right)}^{2}}$$

that was calculated both for the calibration (RMSEC) and validation (RMSECV) sets. Outlier detection was performed using an approach published in [60].

Conventional linear algebra algorithms were used to develop a treatment selection criterion. The two linear PCR equations developed for method LE and method LPI were equated and a hyperplane delineating the methods was obtained. The equation

1 $$Ind\_Full=A_0+\sum_{i=1}^{37}A_ix_i$$

specifies the method to select as follows: if *Ind_Full* < 0, then method LPI is preferred, otherwise method LE. This equation utilizes the full set of 37 parameters, *x*_1_, …, *x*_37_, that must be obtained for each patient, so it is inconvenient in everyday practice. Therefore, we have developed a simplified criterion that depends on only four easily available parameters.

2 $$Ind\_Short={B}_{0}+{B}_{1}\cdot Gender+{B}_{2}\cdot IOP+{B}_{3}\cdot AL+{B}_{4}\cdot ACD$$

Thirty-seven clinical and anatomical parameters included age, gender, spherical equivalent, maximum visual acuity with and without correction, IOP, presence/absence of initial cataract, macular choroidal thickness at 13 points, axial length, anterior chamber depth, lens vault, iris curvature, iris thickness of 750 µm from the scleral spur in nasal and temporal sectors, angle opening distance (AOD500, AOD750), trabecular-iris space area (TISA500, TISA750), and the degree of angle opening according to the Shaffer’s scale in the upper and lower sectors.

## Results and data interpretation

Of 76 patients, 16 were excluded from the study (2 patients, due to the impossibility of identifying the scleral spur at AS-OCT, and 14 patients could not come for examination within 4 weeks after treatment). The remaining 60 PAC patients were examined. The comparative characteristics of the results of LPI and LE are shown in Table 1.

The results showed that after the treatment there was a significant difference in IOP decrease in both groups, but the decrease was greater after LE (Table 1). There was no significant decrease in the number of topical hypotensive drops after LPI, while after LE, there was a statistically significant difference compared to the baseline.

### Verification of similarity of groups 1 and 2

Verification of similarity was performed via testing of two hypothecs. The first one is that group LPI_pre is similar to group LE_pre, and the second one is that group LE_pre is similar to group LPI_pre. The tests were conducted using the DD-SIMCA classifier [28] under a significance value of 0.01. The tests yielded the following values of power of test.

- LE_pre versus LPI_pre, empirical = 1; theoretical = 0.99
- LPI_pre versus LE_pre, empirical = 1; theoretical = 0.98

These outcomes truly confirm the groups’ similarity.

### Building of prediction models for LPI and LE hypotensive effect (ΔIOP)

For both treatment methods, PCR models were built to predict ΔIOP value based on 37 clinical and anatomical parameters described in the section “Study design”.

To predict the results of lensectomy, LE model was built, which uses 2 principal components (PC), where the calibration tolerance RMSEC = 0.79, and the validation tolerance RMSECV = 0.87.

Using the model built for group 1, it is possible to predict the outcome of LE to patients in group 2 and compare it with the actual result obtained with LPI.

**Fig. 1:**
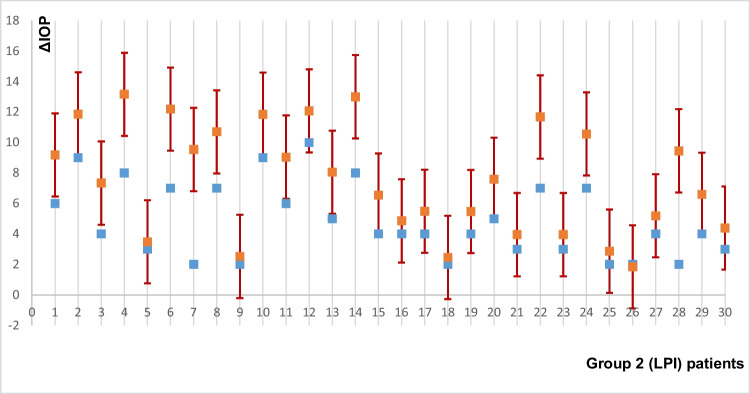
Result of predicting IOP change in the LPI group if these patients had undergone LE; blue marks are actual delta intraocular pressure (ΔIOP) in the LPI; LPI, laser peripheral iridotomy; red marks, predicted ΔIOP in the LPI group in case of lens extraction taking into account the possible modelling error. Intervals- ± 3*RMSEP

Figure 1 shows the hypothetical outcome of IOP change in the LPI group when these patients were treated with LE.

Figure 1 shows that ΔIOP would be hypothetically significantly higher, except for the patients with goniosynechia, in the case of treating LPI patients with LE.

The results of LPI can be predicted in the same way. For this purpose, a PCR model, LPI model, with 2 PCs is used, where the calibration tolerance RMSEC = 0.39, and the validation tolerance RMSECV = 0.41.

**Fig. 2:**
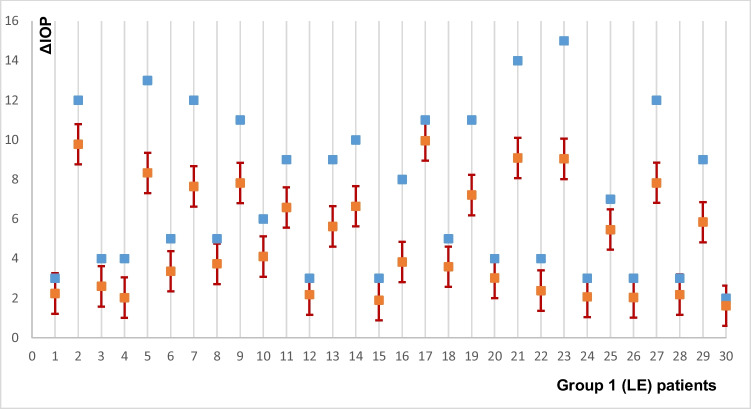
Result of predicting IOP change in the LE group if these patients had undergone LPI; blue marks are actual delta intraocular pressure (ΔIOP) in the LE; LPI, laser peripheral iridotomy; red marks, predicted ΔIOP in the LE group in case of laser peripheral iridotomy taking into account the possible modelling error. Intervals- ± 3*RMSEP

Using this model, it is possible to predict the IOP change that group 1 would have if treated with LPI (Fig. 2).

**Fig. 3:**
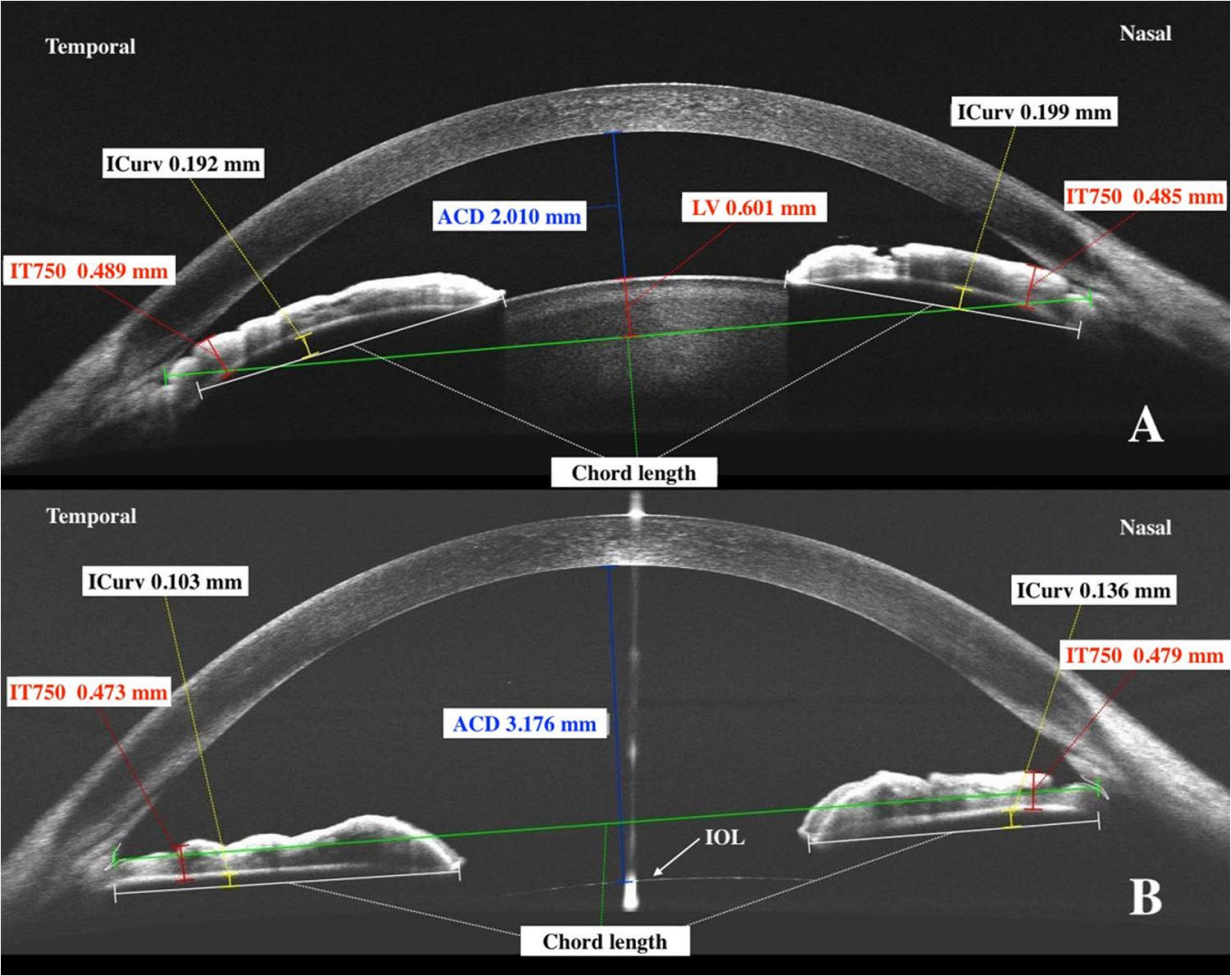
Anterior chamber parameters of a PAC patient with goniosynchia before and after lens extraction; **A** parameters before lens extraction, LE; **B** parameters after LE; ACD anterior chamber depth; LV lens vault; ICurv iris curvature; IT750 iris thickness at 750 µm from scleral spur

Figure 2 shows that for most patients in the LE group, the IOP decrease would be less in LPI, except for some patients with goniosynechia. Figure 3 shows an example of a patient before and after LE, which differed from others in the lensectomy group by the presence of goniosynechia of 60° length in the upper sector. Despite an increase in ACD by 1.166 mm, a decrease in iris curvature by 0.089 mm in the temporal sector and by 0.063 mm in the nasal sector, in IT750 by 0.016 mm and 0.06 mm, respectively, IOP decreased from 24 to 21 mmHg, and this required the prescription of topical hypotensive therapy (1 drop of brimonidine 0.2% 2 times a day).

Predicting the results of treatment using the LE model and the LPI model, it is possible to estimate the IOP decrease in each particular case using both methods and then decide whether this is enough for a particular patient.

### Choice of a treatment method

Using the methodology presented in section “Study design”, the full, Eq. (1), and short, Eq. (2), indicator variables were developed.

The selection of variables was carried out in a standard way [59], in which the importance of a variable was determined by the change in RMSEC and RMSECV values before and after the removal of the variable under study. If both values changed slightly (Fisher’s test, *p* = 0.05), then this variable was removed; otherwise, it was retained.

Table 2 shows the coefficients obtained for the short indicator, *Ind_Short.*

**Table 2:** Coefficients for *Ind_Short*

*B*_0_	Gender	IOP	AL	ACD
16.80	− 0.28	0.24	− 0.65	− 2.36

**Fig. 4:**
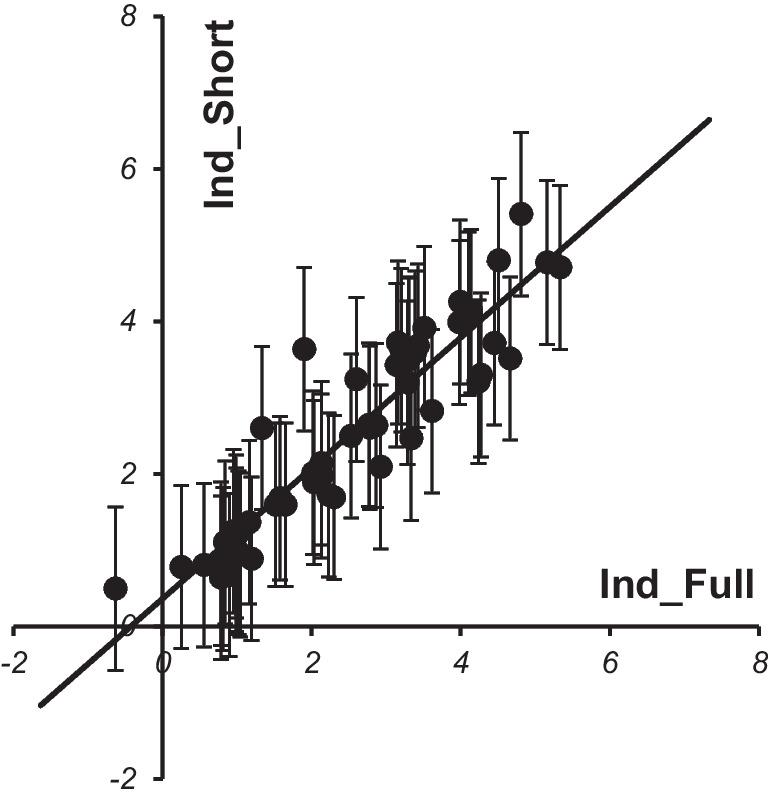
Correlation between values of short and full indicators calculated for patients of both groups

Figure 4 shows the correlation between the full and short indicators, and confirms that the simplified formula given in Eq. (2) can be used in practice. The 0.95 confidence intervals shown in Fig. 4 correspond to the doubled error obtained at the replacement of Ind_Full with Ind_Short. The error value is about 1 that confirms the applicability of the simplified criterion Ind_Short in practice.

The *Ind_Short* result shows the quantitative advantage of one method over the other. For example, if *Ind_Short* = 3, then the IOP decrease in LE will be 3 mm Hg higher than in LPI. If *Ind_Short* =  − 3, then it is recommended to use LPI, because the IOP decrease in LE will be 3 mm Hg less than in LPI. The accuracy of estimation of *Ind_Short* is 1 mm Hg, so the area *Ind_Short* < 1 is recommended to be considered as a “gray zone,” where no method has an advantage.

## Conclusions, expert recommendations, and outlook in the framework of 3PM

The presented study has confirmed the working hypothesis. The proposed approach follows principles of the paradigm change from reactive medical services (applied to clinically established glaucomatous damage) to predictive, preventive, and personalized medicine (3PM/PPPM) applied to vulnerable groups in the population. Great impacts are expected by improving individual outcomes of preventable glaucomatous damage (concretely PACG) accompanied by positive cost-efficacy of advanced medical services to the population (e.g., in the form of innovative screening programs) utilizing predictive disease modelling and treatment algorithms tailored to the personalized patient profile. Essential multi-parametric analysis is implementable by utilizing artificial intelligence (machine learning) in the area.

In the present study, we applied for the first time the quantitative prediction of the hypotensive effect of LE and LPI in PAC based on the machine learning methods using two PCR regression models, LE model and LPI model. We also proposed an innovative workflow based on Eq. (2) that allows creating an individual treatment plan for each patient taking into account the clinical and anatomical parameters.

Moreover, we proposed a short model for choosing a treatment method, which is not inferior to the workflow in terms of its accuracy. This short model is based only on 4 parameters instead of 37, selected with account of the availability of measurements in routine clinical practice: gender, IOP, AL, and ACD (see Table 2).

Comparing the hypothetical ΔIOP in LE in patients in the LPI group with the actual one, we came to the conclusion that most patients would have a greater IOP decrease (Fig. 1). But comparing the hypothetical ΔIOP in LPI in the LE group, in most cases, a less hypotensive effect would be achieved (Fig. 2). However, in the patients with goniosynechia, both LPI and LE are less effective in reducing IOP (Fig. 3). It is known that lens extraction in the presence of goniosynechia does not lead to a decrease in iridotrabecular contact; therefore, in such cases, lensectomy with goniosinechiolysis is necessary [64].

Thus, the use of the proposed workflow based on machine learning allows choosing a treatment method for an individual patient. In addition, the method gives new possibilities for studying the pathogenesis of IOP increase in primary anterior chamber angle closure. Summarized parameters are presented in Table 1.

The limitation of the study is that the presented mathematical models are based on relatively small datasets (60 eyes), which can affect the accuracy of modelling. For further application, it is required to increase the sample size and refine the models.

A multi-parametric analysis to predict glaucomatous damage is an essential approach as demonstrated by several studies [65, 66]**.** Moreover, an advanced PPPM approach applied to affected individuals has been proposed for some types of glaucoma such as the normal-tension glaucoma which otherwise healthy vasospastic individuals are predisposed to [67, 68]. The key tool proposed is the multi-level diagnostics. To this end, for the future application of AI in the area, it is strongly recommended to consider the following:

1. Clinically relevant phenotyping applicable to advanced population screening [69]
2. Systemic effects causing suboptimal health conditions considered in order to cost-effectively protect affected individuals against health-to-disease transition [70–73]
3. Clinically relevant health risk assessment utilizing health/disease-specific molecular patterns detectable in body fluids with high predictive power such as a comprehensive tear fluid analysis [74].

## Supplementary Information

Below is the link to the electronic supplementary material.

Supplementary file1 (DOCX 26 KB)

## Abbreviations

ACA: Anterior chamber angle
ACD: Anterior chamber depth
AI: Artificial intelligence
AL: Axial length
AOD: Angle opening distance
AS-OCT: Anterior segment optical coherence tomography
CT: Choroidal thickness
DD-SIMCA: Data-driven soft independent modelling of class analogies
GON: Glaucomatous optic neuropathy
ICurv: Iris curvature
IOP: Intraocular pressure
IT750: Iris thickness at 750 µm from scleral spur
LE: Lens extraction
LOCS: Lens opacities classification system
LPI: Laser peripheral iridotomy
LV: Lens vault
ML: Machine learning
PACG: Primary angle closure glaucoma
PAC: Primary angle closure
PACD: Primary angle closure disease
PACS: Primary angle closure suspects
PAS: Peripheral anterior synechia
PC: Principal components
PCR: Principal component regression
PCV: Procrustes cross-validation
POAG: Primary open-angle glaucoma
3PM/PPPM: Predictive, preventive, and personalized medicine
RMSE: Root mean squared error
SAP: Static automatic perimetry
SS-OCT: Swept-source optical coherence tomography
TISA: Trabecular-iris space area
UBM: Ultrasound biomicroscopy
