# Supplementary material for: Machine learning–couched treatment algorithms tailored to individualized profile of patients with primary anterior chamber angle closure predisposed to the glaucomatous optic neuropathy
Source: EPMA J. 2023 Aug 17;14(3):527–38. doi: 10.1007/s13167-023-00337-1 (PMC10439872; doi:10.1007/s13167-023-00337-1)
Supplement: Supplementary file 1 — Supplementary file1 (DOCX 26 KB) [file 13167_2023_337_MOESM1_ESM.docx]

**PPPM Innovation Highlights**

**Working hypothesis in framework of 3P medicine**

The present study was initiated due to the lack of literature on the use of mathematical modeling methods based on machine learning in the field of assessing efficacy of PAC treatment. We hypothesized that the multivariate data analysis utilizing machine learning may lead to development of clinically applicable models relevant for improved individual outcomes of the primary angle closure treatment by predicting and preventing glaucomatous damage. For doing this, we suggested a positive dynamics in the intraocular pressure (IOP) after treatment, as the objective measure of a successful treatment. Thirty seven anatomical parameters have been considered by applying artificial intelligence to the prospective study on 30 (LE)+30 (LPI) patients with PAC.

**Relevance for predictive, preventive and personalized medicine**

The presented study has confirmed the working hypothesis. The proposed approach follows principles of the paradigm change from reactive medical services (applied to clinically established glaucomatous damage) to predictive, preventive, and personalized medicine (3PM/PPPM) applied to vulnerable groups in the population. Great impacts are expected by improving individual outcomes of preventable glaucomatous damage (concretely PACG) accompanied by positive cost-efficacy of advanced medical services to the population (e.g. in form of innovative screening programs) utilizing predictive disease modelling and treatment algorithms tailored to the personalized patient profile. Essential multi-parametric analysis is implementable by utilizing artificial intelligence (machine learning) in the area.

**Innovation beyond the state of the art**

In the present study, we applied for the first time the quantitative prediction of hypotensive effect of LE and LPI in PAC based on the machine learning methods using two PCR regression models, LE-model and LPI-model. We also proposed an innovative workflow based on Equation (2) that allows creating an individual treatment plan for each patient taking into account the clinical and anatomical parameters.

Moreover, we proposed a short model for choosing a treatment method, which is not inferior to the workflow in terms of its accuracy. This short model is based only on 4 parameters instead of 37, selected with account of the availability of measurements in routine clinical practice: gender, IOP, AL, and ACD (see Table 1).

Comparing the hypothetical ΔIOP in LE in patients in the LPI group with the actual one, we came to the conclusion that most patients would have a greater IOP decrease (Fig. 1). But comparing the hypothetical ΔIOP in LPI in the LE group, in most cases, a less hypotensive effect would be achieved (Fig. 2). However, in the patients with goniosynechia, both LPI and LE are less effective in reducing IOP (Fig. 3). It is known that lens extraction in the presence of goniosynechia does not lead to a decrease in iridotrabecular contact; therefore, in such cases, lensectomy with goniosinechiolysis is necessary.

Thus, the use of the proposed workflow based on machine learning allows choosing a treatment method for an individual patient. In addition, the method gives new possibilities for studying the pathogenesis of IOP increase in primary anterior chamber angle closure. Summarized parameters are presented in the Table 2.

The key-tool proposed is the multi-level diagnostics. Finally, for the future application of AI in the area expert recommendations are provided.
